# Protein Expression Analysis and Functional Characterization of Sorcin in Gallbladder Cancer

**DOI:** 10.3390/cells15080678

**Published:** 2026-04-12

**Authors:** Vaishali Jain, Neeraj Saklani, Srishti Kawatra, Puja Sakhuja, Surbhi Goyal, Anil Kumar Agarwal, Parveen Kumar, Fouzia Siraj, Poonam Gautam

**Affiliations:** 1 ICMR-Centre for Cancer Pathology (Formerly a Part of ICMR-National Institute of Pathology), Safdarjung Hospital Campus, New Delhi 110029, India; vaishalijain2503@gmail.com (V.J.); neerajsaklani0808@gmail.com (N.S.); srishtikawatra97@gmail.com (S.K.); parveenkumarxyz@gmail.com (P.K.); fouziasiraj2009@gmail.com (F.S.); 2 Faculty of Health Sciences, Manipal Academy of Higher Education (MAHE), Manipal 576104, India; 3 Department of Pathology, Govind Ballabh Pant Institute of Postgraduate Medical Education and Research (GIPMER), New Delhi 110002, India; dr.surbhi4you@gmail.com; 4 Department of Surgery, Govind Ballabh Pant Institute of Postgraduate Medical Education and Research (GIPMER), New Delhi 110002, India; aka.hpb@gmail.com

**Keywords:** gallbladder cancer, Sorcin, immunohistochemistry, epithelial–mesenchymal transition, therapeutic target

## Abstract

**Highlights:**

**What are the main findings?**

- Sorcin (*SRI*) is significantly overexpressed in gallbladder cancer tissues compared to gallstone disease controls, with positive expression observed in 67% of tumors compared to controls.
- siRNA-mediated knockdown of *SRI* in gallbladder cancer cells suppresses proliferation, migration, invasion, and epithelial–mesenchymal transition.

**What are the implications of the main findings?**

- *SRI* functions as an oncogenic driver in gallbladder cancer progression.
- Targeting *SRI* may represent a novel therapeutic strategy for gallbladder cancer, warranting further validation in preclinical models.

**Abstract:**

Gallbladder cancer (GBC) is an aggressive malignancy with limited treatment options and poor clinical outcomes. Identifying novel molecular targets is critical for improving therapeutic strategies. Sorcin (*SRI*), a calcium-binding protein implicated in tumor progression, has not been comprehensively investigated in GBC. *SRI* expression was analyzed by immunohistochemistry (IHC) in a large cohort of gallstone disease (GSD) controls (*n* = 85) and GBC tissues (*n* = 85). Functional assays, including cell proliferation, wound healing, transwell invasion, and Western blot analyses of epithelial–mesenchymal transition (EMT) markers, were performed in the NOZ GBC cell line following siRNA-mediated *SRI* knockdown. IHC revealed that 67% of GBC cases exhibited positive staining whereas all the GSD cases exhibited negative staining of *SRI*, demonstrating a significant upregulation of *SRI* in GBC (*p* < 0.001). *SRI* knockdown resulted in reduced proliferative capacity and markedly impaired migration and invasion. Further, *SRI* knockdown decreased vimentin levels, indicating suppression of EMT. *SRI* is significantly overexpressed in GBC and promotes key oncogenic traits, including proliferation, migration, invasion, and EMT. These findings highlight *SRI* as a potential therapeutic target in GBC. Further validation in animal models may facilitate translation into clinical applications.

## 1. Introduction

Gallbladder carcinoma (GBC) is the sixth most common malignant aggressive tumor of the gastrointestinal tract and is often diagnosed at advanced stages [1,2]. It is more prevalent in Asian countries, which account for nearly 70% of newly diagnosed GBC cases worldwide [2]. Complete surgical resection remains the most effective potentially curative approach in carefully selected patients. Systemic therapy is an option for patients with advanced or metastatic GBC [3]. Despite current standard therapies, patient outcomes remain poor, with a median overall survival of 19 months and a 5-year survival rate of 28.8% [4]. Given the poor treatment outcomes, there is an urgent need to identify novel therapeutic targets for GBC.

Sorcin (*SRI*) is a soluble resistance-related calcium-binding protein and is located in chromosome 7q21 [5]. It exists in two main isoforms: the 22 kDa isoform and the 18 kDa isoform. The 22 kDa isoform is predominantly found in the cytosol, while the 18 kDa isoform specifically localizes to the mitochondria. *SRI* comprises two distinct domains: a flexible and hydrophobic N-terminus rich in Gly/Pro residues, and a C-terminal domain that binds calcium through five EF-hand motifs. The EF-hand motif is a common structural feature used by proteins to interact with calcium ions [6]. It is found in various mammalian tissues including cardiac muscle, skeletal muscle, the brain and kidney. In normal tissue, *SRI* regulates multiple target proteins and plays a key role in calcium homeostasis by modulating channels, pumps, and exchangers. It inhibits calcium release from the endoplasmic reticulum by binding to ryanodine receptors (RyRs), thereby suppressing Ca^2+^ signaling. In cardiac cells, *SRI* strongly inhibits RyR activity, reducing both spontaneous and Ca^2+^-induced Ca^2+^ release (CICR) [7]. In addition to normal tissues, *SRI* is found to be overexpressed in variety of cancers including breast cancer, hepatocellular carcinoma (HCC), colorectal cancer, gastric cancer, lung cancer, ovarian cancer, leukemia, and myeloma [8].

*SRI* overexpression is associated with multidrug resistance against different chemotherapeutic drugs including 5-fluorouracil, vincristine cisplatin, gemcitabine, doxorubicin, etoposide, paclitaxel, etc. [8]. Its contribution to multiple-drug resistance (MDR) is through regulation of ATP-binding cassette sub-family B member 1 (ABCB1) or (MDR1/P-glycoprotein), a broad-substrate ATP-dependent efflux pump that exports many chemotherapeutics out of cancer cells [8]. The overexpression of *SRI* correlates closely with ABCB1 overexpression in multidrug-resistant cell lines and primary tumors [7,9]. At the mechanistic level, *SRI* promotes drug resistance by activating protein kinase A (PKA), which in turn increases phosphorylation of the transcription factor Cyclic AMP-responsive element-binding protein 1 (CREB1). When *SRI* is silenced, CREB1 phosphorylation is reduced, resulting in lower ABCB1 mRNA and protein levels and a decline in drug efflux activity, as evidenced by rhodamine-123 and doxorubicin efflux assays [7,9,10] which ensure sustained pump activity even under chemotherapeutic stress.

*SRI* promotes tumor progression by enhancing invasion and migration; inhibits mitochondrial apoptosis via interaction with tumor necrosis factor type 1 receptor-associated protein (TRAP1); and regulates apoptotic proteins (Bax, Bcl-2, caspase-3) through signaling pathways including extracellular signal-regulated kinase (ERK), signal transducer and activator of transcription 3 (STAT3), and phosphatidylinositol 3-kinase (PI3K)/protein kinase B (AKT) [11]. It inhibits the action of caspase-3, caspase-12, and 78 kDa glucose-regulated protein (GRP78)/binding immunoglobulin protein (BiP), thereby inhibiting apoptosis [8]. *SRI* also regulates many proteins associated with tumorigenesis including nuclear factor kappa-light-chain-enhancer of activated B cells (NF-κB), STAT3, AKT, ERK1/2, inositol 1,4,5-trisphosphate receptor (IP3R), vascular endothelial growth factor (VEGF), and matrix metalloproteinase (MMP) [5]. Several reports reveal that *SRI* mitigates tumor invasion and metastasis by regulating the levels of Cathepsin Z (CTSZ), STAT-3 and MMP2 and MMP9 [8]. Hu et al. reported *SRI* silencing impedes proliferation and metastasis of breast cancer cells, suppressing angiogenesis by decreasing the expression of VEGFs in vivo and reducing the cancer stem cell (CSC) population [12]. A recent study by Li et al. revealed that *SRI* is highly expressed in hepatocarcinoma cells and its ablation inhibited the growth, migration and invasion of HCC cells by inducing pyroptosis via interaction with the NLRP3 inflammasome [13]. In our previous study [14], we observed the overexpression of *SRI* and its association with LN metastasis in GBC.

Despite emerging evidence implicating *SRI* in multidrug resistance and tumor progression in various cancers, its clinical relevance and functional role in GBC remain largely unexplored. In the present study, we aimed to investigate the expression pattern of *SRI* in GBC and gallstone disease (GSD) (non-tumor control) tissues using immunohistochemistry (IHC) in a larger patient cohort to assess its potential association with GBC. Furthermore, we performed *SRI* knockdown (KD) experiments in the GBC cell line, NOZ, to elucidate the functional significance of *SRI* in regulating cell proliferation, invasion, and migration in GBC.

## 2. Materials and Methods

### 2.1. Patient Samples

The patients were enrolled at the Govind Ballabh Pant Institute of Postgraduate Medical Education & Research (GIPMER), New Delhi, following approval from the Institutional Human Ethics Committees of both Maulana Azad Medical College (IEC No. (F.1/IEC/MAMC/(80/08/2020/No. 314) and the ICMR National Institute of Pathology, New Delhi (IEC No. NIP-IEC/10-12-19/06). All procedures were performed in accordance with the Declaration of Helsinki, and informed consent was obtained from all participants.

In this prospective study, we collected formalin-fixed paraffin-embedded (FFPE) tissue blocks from a cohort of 85 + 85 patients. It was composed of two groups: a control group comprising 85 cases of GSD with no evidence of dysplasia and GBC group with 85 cases. Staging was performed according to the American Joint Committee on Cancer (AJCC) 8th edition guidelines [15], based on patient clinical data, histopathological evaluation, and imaging. Inclusion criteria were: age ≥18 years; and histologically confirmed GBC adenocarcinoma (for cases) or gallstone disease without malignancy (for controls). Exclusion criteria included: patients who were morbidly ill; and those who had previously received treatment for cancer. Clinicopathological data of these subjects are detailed in Table 1. Clinical parameters such as TNM, stage, grade, white cell count, liver enzymes (AST/ALT/ALP), bilirubin and co-morbidities (jaundice, diabetes mellitus, hypertension, loss of appetite and loss of weight) for the GBC patients and control groups as available (~45%) are provided in Supplementary Table S1.

### 2.2. Immunohistochemistry Analysis

IHC was performed on FFPE tissues using individual tissue sections from non-tumor controls, GSD cases (*n* = 85) and GBC cases (*n* = 85) (Table 1) to analyze the expression of *SRI* protein. IHC analysis was performed as described earlier by Jain et al. [14]. In brief, after deparaffinization and rehydration of FFPE tissue sections, antigen retrieval was performed by immersing the slide in antigen retrieval buffer (20 mM Tris buffer, pH 9.0) at 90 °C for 20 min. Endogenous peroxidases were blocked with 0.03% hydrogen peroxide, and nonspecific binding was blocked with protein-blocking reagent. Sections were then incubated for 1 h at RT with primary antibody against *SRI* (dilution 1:3000, catalog no. PA5-23,143, Thermo Fisher Scientific, Waltham, MA, USA) followed by incubation with PolyExcel PolyHRP for 40 min at RT. Tissue sections were then incubated with Stunn DAB working solution for 5 min at RT (PathnSitu Biotechnologies, Pleasanton, CA, USA). Sections were counter stained with Mayer’s hematoxylin, dehydrated and images were taken under the microscope. The distribution of staining and staining intensity across the section was observed under the microscope. The scoring criteria were based on both staining intensity and distribution. For *SRI*, a 2 + or higher intensity with ≥10% distribution was considered as ‘positive’, while 1 + positivity or <10% distribution was considered as ‘negative’. The data was analyzed for both nuclear and cytoplasmic expression of *SRI*. IHC data analysis was done by two independent pathologists. The statistical analysis (Fisher’s exact test, two-sided) was performed using GraphPad Prism version 8 to study the correlation of *SRI* expression among GBC vs. GSD. Further, the correlation of *SRI* expression with clinicopathological features such as tumor grade [poorly differentiated adenocarcinoma (PDAC) vs. well and moderately differentiated adenocarcinoma (WDAC + MDAC)] and lymph node (LN) metastasis (LN positive vs. LN negative GBC) was analyzed. A *p*-value less than 0.05 indicated statistical significance.

### 2.3. Cell Culture

Human GBC cell line, NOZ, was obtained from Japanese Collection of Research bioresources Bank, Osaka, Japan. The cells were cultured in William’s media supplemented with 10% heat-inactivated fetal bovine serum (FBS) and 1% antibiotics in a 5% carbon dioxide and 37 °C atmosphere. Cell line was routinely tested for mycoplasma by PCR.

### 2.4. siRNA Transfection

Predesigned siRNA against *SRI* (cat no. 4392420, Thermo Fisher Scientific, Waltham, MA, USA) was used to transfect cells. Cells in logarithmic growth phase were trypsinized, counted, and seeded in 24-well plates to ensure 50% cell confluence on the next day for transfection. Transfection of cells with oligonucleotides was performed using Lipofectamine™ RNAimax reagent in line with the manufacturer’s instructions (Invitrogen, Carlsbad, CA, USA). Briefly, NOZ cells were cultured in 24-well plates at a density of 5 × 10^4^ cells per well. Then, the cells were transfected with either siRNA against *SRI* or scramble control (si-control) at a concentration of 2.5 picomoles using Lipofecatmine RNAimax. The transfection medium was replaced with fresh complete medium after 24 h. Transfection efficiency was evaluated using Western blot analysis.

### 2.5. Protein Extraction

After 72 h of siRNA treatment, the cells were collected and lysed in modified RIPA with 1% protease inhibitor cocktail followed by sonication (Biologics 3000MP, BioLogics Inc., Manassas, VA, USA) with three bursts of 10 s each and 10 s of pause interval at 4 °C for protein extraction. After centrifugation at 14,000× *g* for 30 min, the protein concentration of harvested supernatant was determined by the Bradford assay.

### 2.6. Western Blotting

The protein lysates (15 μg/lane) were separated by 12% SDS polyacrylamide gels and then blotted onto polyvinylidene difluoride (PVDF) membranes. The blots were blocked with 5% skimmed milk powder in TBST [1× tris buffered saline (10 mM Tris-Cl, pH 7.4 and 30 mM NaCl) with 0.05% Tween 20 and 0.005% Triton-X-100] at RT for 1 h. The blots were then incubated with primary antibodies against *SRI* (1:1000, Thermo Fisher Scientific, Waltham, MA, USA), vimentin (1:1000, Thermo Fisher Scientific, Waltham, MA, USA) and N-cadherin (1:1000, Thermo Fisher Scientific, Waltham, MA, USA) at 4 °C overnight. Secondary antibodies against rabbit IgG (1:20,000, Thermo Fisher Scientific, Waltham, MA, USA) for *SRI* and mouse IgG (1:20,000, Thermo Fisher Scientific, Waltham, MA, USA) for vimentin and N-cadherin were used at RT for 1 h. The blots were developed using the enhanced chemiluminescent (ECL) kit (Millipore, NJ, USA). The images were acquired using Chemidoc MP imager and immunoblots were analyzed using Image Lab 4.1 software (Bio-Rad Laboratories, Hercules, CA, USA). Densitometric analysis of the specific band showing reactivity was performed to get relative expression of target proteins in control vs si-control and si-*SRI*. The maximum density of control was used to calculate the fold change in expression of si-*SRI*. The protein load was normalized based on the total density of proteins in each sample. The expression of β-actin was used as a loading control.

### 2.7. Cell Proliferation Analysis

Cancer cell proliferation was evaluated using the Cell Counting Kit (CCK)-8 assay (cat. no. ab228554; Abcam, Cambridge, UK). NOZ cells were seeded in 24-well plates (5.0 × 10^3^ cells/well) and transfected with *SRI* siRNA or control siRNA for 0, 24, 48, 72 and 96 h at 37 °C. Then, CCK-8 reagent (30 µL) was added to each well. Each plate was incubated for 2 h at 37 °C, and the absorbance at 450 nm was measured. The experiment was performed in triplicates. Images were taken using a microscope.

### 2.8. Wound Healing Analysis

Equivalent numbers of NOZ cells (5 × 10^5^) were seeded in 24-well plates, and wounds were created using 10 µL pipette tip in the cells covering the plate. After the cells were extensively rinsed with Dulbecco’s Phosphate-Buffered Saline to remove cellular debris, NOZ cells were cultured in complete growth media. The scratched area was captured by a light microscope (magnification, ×10) and the area gap was measured using ImageJ software v1.4.3.67 [16] by finding the edges of the wound. The experiment was performed in triplicates.

### 2.9. Cell Invasion Analysis

A cell invasion assay was conducted by using BD BioCoat Matrigel invasion chambers BD Biosciences, San Jose, CA, USA). After the rehydration of the chambers, 50,000 cells suspended in 150 µL FBS-free media were seeded onto the Matrigel over the upper chamber. Media containing 10% FBS were added to the bottom of the lower chamber. After incubation for 48 h, the non-invading cells were removed from the upper surface of the membrane, whereas the cells that invaded through and adhered to the bottom of the membrane were fixed with 4% paraformaldehyde and stained with crystal violet for 10 min. The bound crystal violet was eluted by adding 400 µL of 33% acetic acid into each insert and shaking for 20 min. The eluent from the lower chamber was transferred to a 96-well clear microplate and the absorbance at 590 nm was measured using a plate reader. The experiment was performed in triplicates.

### 2.10. Statistical Analysis

All experiments were performed in triplicates, and the data are expressed as the mean ± SEM (standard error of mean). All comparisons to the KD samples were performed using a two-tailed, independent samples t-test. Statistical significance was determined at *p*-value < 0.05.

## 3. Results

### 3.1. SRI Expression by Immunohistochemistry Analysis

**Figure 1:**
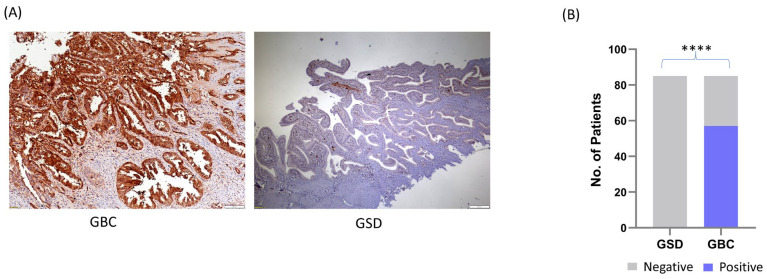
IHC analysis to study the expression of *SRI* in the GBC cases and control group. (**A**) Representative IHC images showing the expression of *SRI* in GBC and GSD (non-tumor controls). IHC was performed on formalin-fixed paraffin-embedded (FFPE) individual tissue sections of 85 controls (GSD cases with no dysplasia), and 85 GBC cases. The IHC results showed that the expression of *SRI* was found to be ‘positive’ in 57 GBC cases (67%) while all of the GSD cases showed negative expression. (**B**) The statistical (****) analysis between GBC and GSD showed a significant difference in *SRI* expression (*p* value < 0.0001). The scale bar (500 µm) is shown as white line. The method for IHC scoring is shown in methodology, Section 2.8. GSD—gallstone disease, GBC—gallbladder carcinoma, *SRI*—Sorcin, IHC—immunohistochemistry.

We performed IHC analysis to study the expression of *SRI* in GSD (non-tumor control) and GBC. The expression of *SRI* was found to be ‘negative’ in 100% GSD (*n* = 85). *SRI* expression was found to be ‘positive’ in 57 GBC cases (67%) and negative in 28 GBC cases (33%). Figure 1A shows the representative IHC images of controls and GBC. The statistical analysis between GBC vs GSD showed a significant difference in *SRI* expression (*p* value < 0.0001) (Figure 1B). The *SRI* expression did not show any significant correlation with clinicopathological features such as the tumor grade and lymph node metastasis.

### 3.2. SRI Knockdown in NOZ Cells

**Figure 2:**
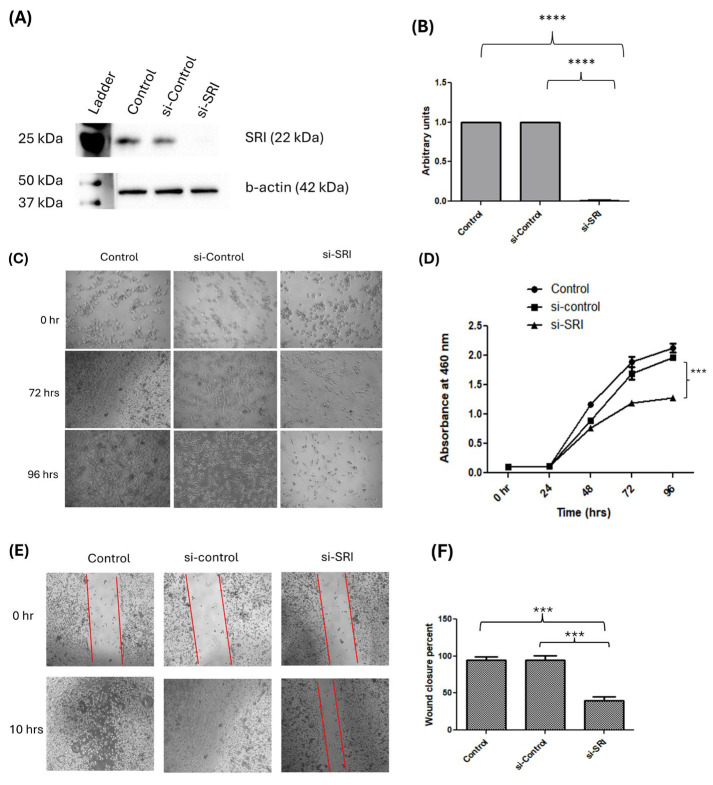
Silencing of *SRI* gene using siRNA-mediated gene knockdown and functional characterization. (**A**) Western blot image of *SRI* knockdown in control, si-control and si-*SRI*. The full-length blot images are given in Supplementary Figure S1. (**B**) Bar diagram depicting densitometric analysis revealing the transfection efficiency of 99% ± 0.27% (*p* < 0.0001). (**C**) The cell proliferation capacity of NOZ cells after *SRI* knockdown was analyzed and the representative image shows NOZ cells (magnification—4×) without treatment and after treatment with si-control and si-*SRI* at 0, 72 and 96 h. (**D**) Graph showing significant reduction (*p* < 0.001) in cell proliferation in si-*SRI* GBC cells as compared to si-controls after 72 h and 96 h by CCK-8 assay. (**E**) Representative image showing the migration capacity of NOZ cells in wound healing assays after *SRI* knockdown. The wounded area was photographed under phase contrast microscope at 0 and 10 h after scratching. The area of the wound was measured at 0 and 10 h using ImageJ software (magnification—4×). (**F**) Bar graph representing wound healing ability of *SRI* knockdown cells after 10 h after inducing the scratch. *SRI* gene knockdown significantly suppressed the migratory ability of NOZ cells by 60%. Data are representative of three independently performed experiments and are expressed as mean ± SEM. *SRI*—Sorcin, CCK-8—Cell Counting Kit-8, GBC—gallbladder cancer. *** *p* < 0.001, **** *p* < 0.0001.

To assess the role of *SRI* in GBC progression, we carried out *SRI* KD in GBC cell lines, NOZ. The transfection efficiency was evaluated using Western blot analysis. Our results indicated that *SRI* protein expression was successfully reduced by 99% ± 0.27% (*p* < 0.0001) (Figure 2A,B).

### 3.3. SRI Knockdown Reduces Proliferative Potential in NOZ Cells

CCK8 proliferation assay was carried out to analyze the effect of reducing *SRI* expression on the proliferative capacity of NOZ cells. *SRI* KD cells showed a significant reduction in proliferation in comparison to si-control cells. Overall, we found a 31.85% ± 3.88% (*p* < 0.01) and 37.70% ± 0.24% (*p* < 0.001) reduction in cell proliferation capacity in *SRI* knockdown cells after 72 h and 96 h respectively. However, no significant difference was observed in the proliferative potential of si-control cells in comparison to the control NOZ cells (Figure 2C,D).

### 3.4. SRI Knockdown Reduces the Migratory Potential of NOZ Cell

To assess the role of *SRI* in the migration of cancer cells, wound healing assay was carried out. Representative images of time points 0, 8 and 10 h for NOZ cells are illustrated in Figure 2E. We observed that the cells transfected with si-*SRI* migrate at a remarkably decreased rate when compared with the control and si-control cells. NOZ control and si-control cells had closed the gap within 10 h; however, the gap remained open in the *SRI* KD cells. This confirmed that the reducing *SRI* expression in the NOZ cell line resulted in decreased migratory potential. The gap area was calculated using ImageJ software (Figure 2F).

### 3.5. SRI Knockdown Reduces the Invasive Potential of NOZ Cells

**Figure 3:**
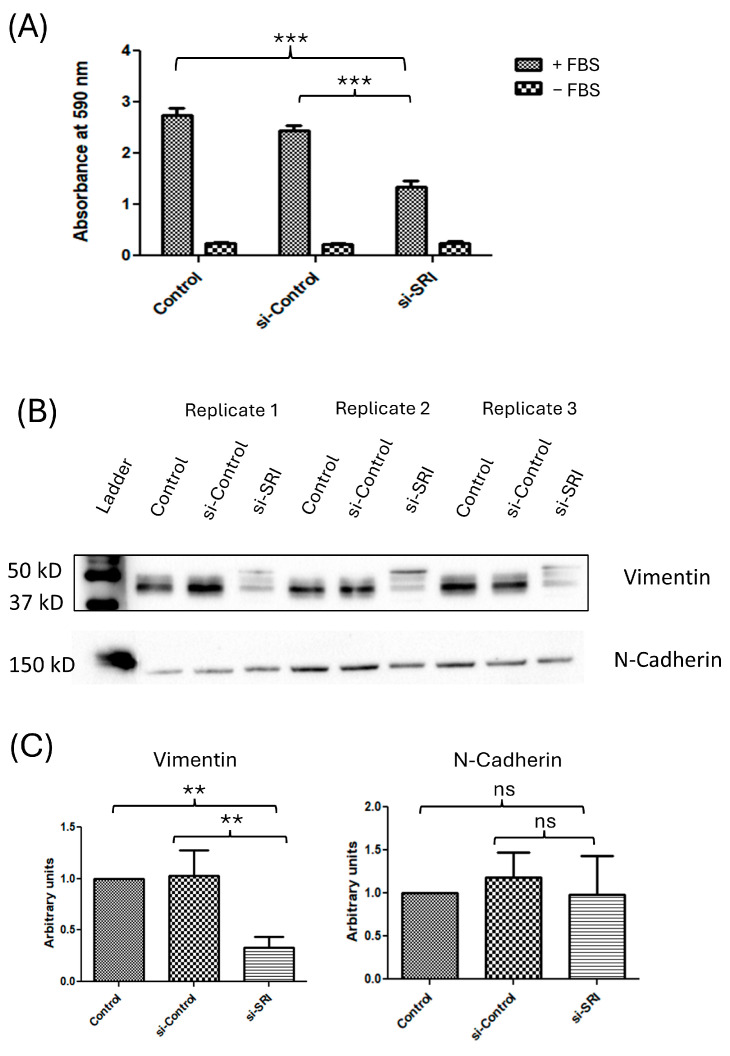
Invasion potential of the NOZ cells after SRI knockdown and analysis of EMT markers. (**A**) Bar graph showing comparative invasion potential of the NOZ cells using Matrigel Transwell Invasion Assay. SRI gene knockdown significantly suppressed the invasion potential of NOZ by 40% (*p* < 0.001). The data are representative of three independently performed experiments and are expressed as mean ± SD. (**B**) Expression of EMT markers, vimentin and N-cadherin, after SRI knockdown using Western blot analysis. (**C**) Band intensity analysis shows knockdown of SRI leads to 2-fold downregulation of mesenchymal marker vimentin while no significant change in expression was observed for N-cadherin. The full-length blot images are given in Supplementary Figure S2. SRI—Sorcin, EMT—epithelial–mesenchymal transition. ** *p* < 0.01, *** *p* < 0.001; ns, not significant.

To study the invasive potential of *SRI* knockdown in NOZ cells, transwell Matrigel assay was used. *SRI* KD cells exhibited 40% decreased invasive potential as compared to control and si-control (*p* < 0.0001) (Figure 3A).

### 3.6. SRI Enhances Metastasis by Promoting EMT

Expression levels of the mesenchymal marker vimentin and epithelial marker N-cadherin were measured using Western blot. Western blot confirmed that *SRI* knockdown resulted in a significant decrease in the expression of vimentin (2.5-fold, *p* < 0.0001). No significant change in expression was observed for N-cadherin. These results indicated that *SRI* enhances metastasis by facilitating epithelial–mesenchymal transition (EMT) by regulating vimentin levels (Figure 3B,C).

## 4. Discussion

Radical resection of the gallbladder along with chemotherapy remains the standard treatment for GBC; however, patient outcomes continue to be poor. Given these suboptimal treatment outcomes, identifying novel molecular targets is essential to improving therapeutic strategies for GBC. In our earlier study, we employed an Isobaric Tags for Relative and Absolute Quantitation (iTRAQ-based quantitative proteomics approach to analyze the differential proteome in lymph node metastatic GBC and *SRI* emerged as a strong candidate [14], prompting us to investigate its expression in a larger tissue cohort using IHC, and to functionally validate its role in vitro. In the present study, we comprehensively evaluated the clinical relevance and functional role of *SRI* in GBC.

IHC analysis in FFPE tissue samples demonstrated a striking overexpression of *SRI* between GBC and GSD (non-tumor control). Approximately 65% of GBC cases showed positive staining, confirming a significant upregulation of *SRI* in malignant tissues while all GSD samples (100%) were *SRI*-negative. Earlier, we reported a significant association of *SRI* with LN-positive GBC in comparison to LN-negative GBC (total sample size, *n* = 30); however, the analysis with the larger cohort in the present study (total sample size, *n* = 85) showed no significant association of *SRI* expression with LN-positive GBC. Overall, *SRI* is a tumor-associated protein and may be used as a tissue-based marker for the confirmation of malignancy in GBC.

To establish its biological significance, we performed functional assays using the NOZ GBC cell line. Efficient *SRI* knockdown (~99%) enabled us to evaluate its contribution to tumorigenesis. *SRI* silencing significantly reduced cell proliferation, migration, and invasion, as demonstrated by CCK-8, wound-healing, and transwell assays. The delayed wound closure observed in *SRI*-deficient cells further supports the role of *SRI* in enhancing motility. These findings clearly indicate that *SRI* promotes oncogenic traits in GBC cells. Mechanistically, *SRI* is reported to influence metastasis through the modulation of EMT in colorectal cancer [17]. *SRI* knockdown led to a significant reduction in vimentin expression, consistent with a shift toward an epithelial phenotype and decreased migratory potential. However, no significant change was observed in N-cadherin levels, suggesting that *SRI* regulates EMT via vimentin. Ling et al. showed an association between *SRI* and Annexin A7 and reported that the overexpression of these proteins promotes EMT in HCC. They reported the downregulation of vimentin and upregulation of E-cadherin after *SRI* knockdown [18]. Similar EMT-related roles for *SRI* have been reported in breast, colorectal, ovarian, and leukemia models, where *SRI* modulates *VEGF*s, *MMP*s, NF-κB, *ERK1/2*, *Akt*, *STAT3*, and caspase signaling pathways [8].

The present study reports the overexpression of *SRI* in GBC in comparison to controls. Functional studies strongly suggest that *SRI* is an important promoter of GBC progression, contributing to proliferation, invasion, migration, and EMT. The integration of IHC evidence with mechanistic in vitro assays underscores its potential as a tissue-based tumor biomarker and therapeutic target.

High-throughput analysis at the transcript and protein level after siRNA knockdown is needed for a better understanding of the molecular mechanism of *SRI* in GBC. Given the limited treatment options for GBC, targeting *SRI* or its downstream pathways may represent a promising avenue for therapeutic development. Naturally occurring compounds (Dihydromyricetin [19], triptolide [20]) inhibiting *SRI* activity in cancer have been reported. Future studies should explore the detailed functional analysis of *SRI* in vivo and assess the efficacy of *SRI* inhibitors independently and in combination with the available therapeutic drugs.

## Figures and Tables

**Table 1:** Clinicopathological parameters of the patients enrolled in the study.

Participants	Total Number	Number of Males	Number of Females	Median Age (Years)	Age Range (Years)
Total GBC cases	85	60	25	53	27–75
Stages ^#^					
GBC, Stage 0	1	0	1	NA	NA
GBC, Stage I	8	2	6	54.5	38–65
GBC, Stage II	20	6	14	54	36–70
GBC, Stage III	32	10	22	53.5	27–75
GBC, Stage IV	18	4	14	50	27–65
Histological grade					
Well-differentiated (G1)	16	4	12	50.5	27–70
Moderately differentiated (G2)	56	16	40	54	27–75
Poorly differentiated (G3)	13	5	7	55	38–72
Lymph Node (LN) status ^#^					
LN Negative	37	11	26	55	36–72
LN Positive	42	11	31	50.5	27–75
Controls					
GSD cases	85	21	64	40	18–75

^#^ Stage and LN status could not be defined for 6 GBC cases; NA—not applicable.

## Data Availability

The original contributions presented in this study are included in the article/Supplementary Materials. Further inquiries can be directed to the corresponding authors.

## Supplementary Materials

The following supporting information can be downloaded at: https://www.mdpi.com/article/10.3390/cells15080678/s1, Figure S1: Full-length blot images of Figure 2A for the expression of *SRI* in the control, si-control and si-*SRI*. (A) SDS-PAGE image showing protein profile of cell lysate. Total density was used for normalization of protein load of cell lysate from control, si-control and si-*SRI*. (B) Western blot image showing expression of b-actin used as loading control. (C) Western blot image showing expression of *SRI* in control, si-control and si-*SRI*. The experiment was performed in triplicates. The cropping of the blot images is indicated with red dashed line; Figure S2: Full-length blot images of Figure 3B for the expression of (A) vimentin and (B) N-cadherin in the control, si-control and si-*SRI*. The experiment was performed in triplicates. The cropping of the blot images is indicated with red dashed line; Table S1: Clinical parameters of patients.

## Abbreviations

The following abbreviations are used in this manuscript:

GBC	Gallbladder cancer
GSD	Gallstone disease
*SRI*	Sorcin
FFPE	Formalin-fixed paraffin-embedded
IHC	Immunohistochemistry
PCR	Polymerase Chain Reaction
EMT	Epithelial–Mesenchymal Transition
