# Supplementary material for: Protein Expression Analysis and Functional Characterization of Sorcin in Gallbladder Cancer
Source: Cells. 2026 Apr 12;15(8):678. doi: 10.3390/cells15080678 (PMC13115428; doi:10.3390/cells15080678)
Supplement: Supplementary file 1 [file cells-15-00678-s001.zip › Supplementary Images.pdf]

Supplementary Images

(A)

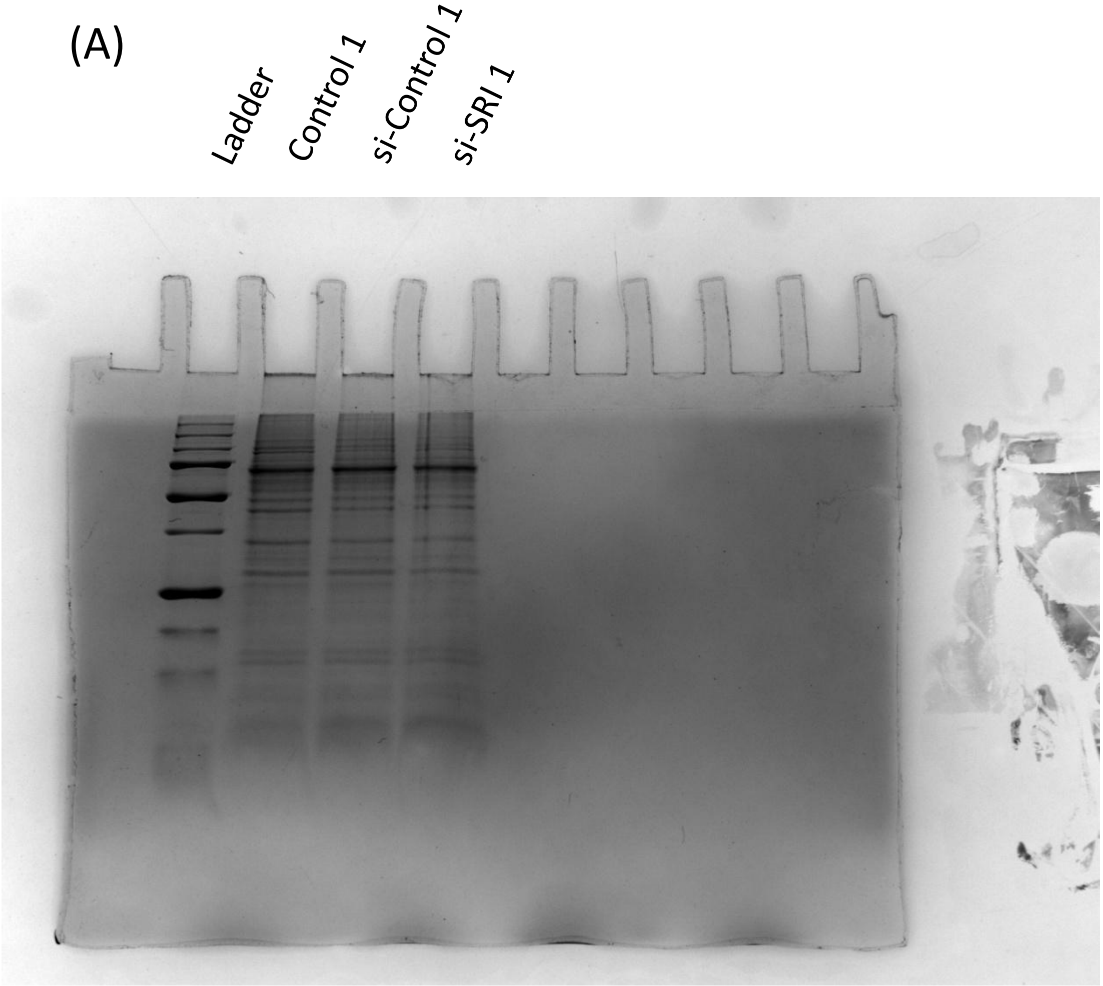

(B)

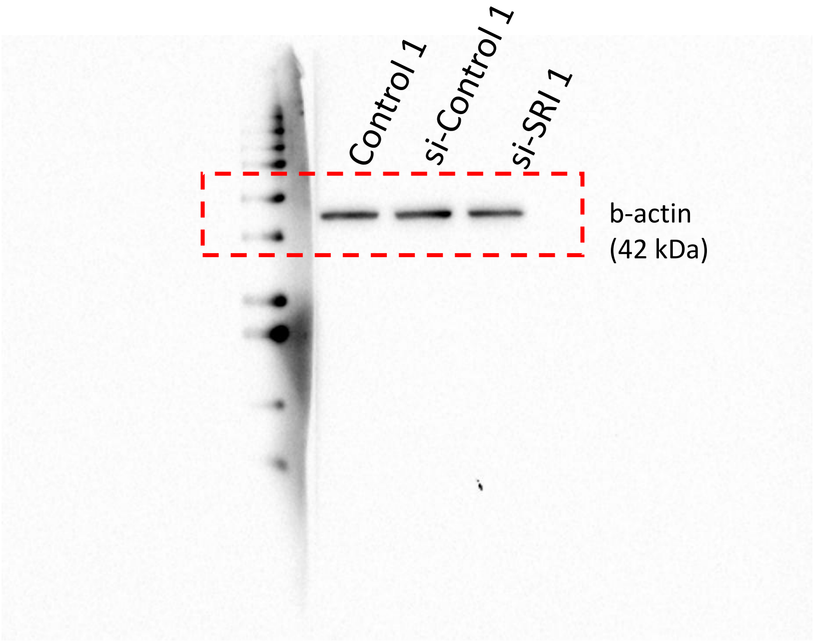

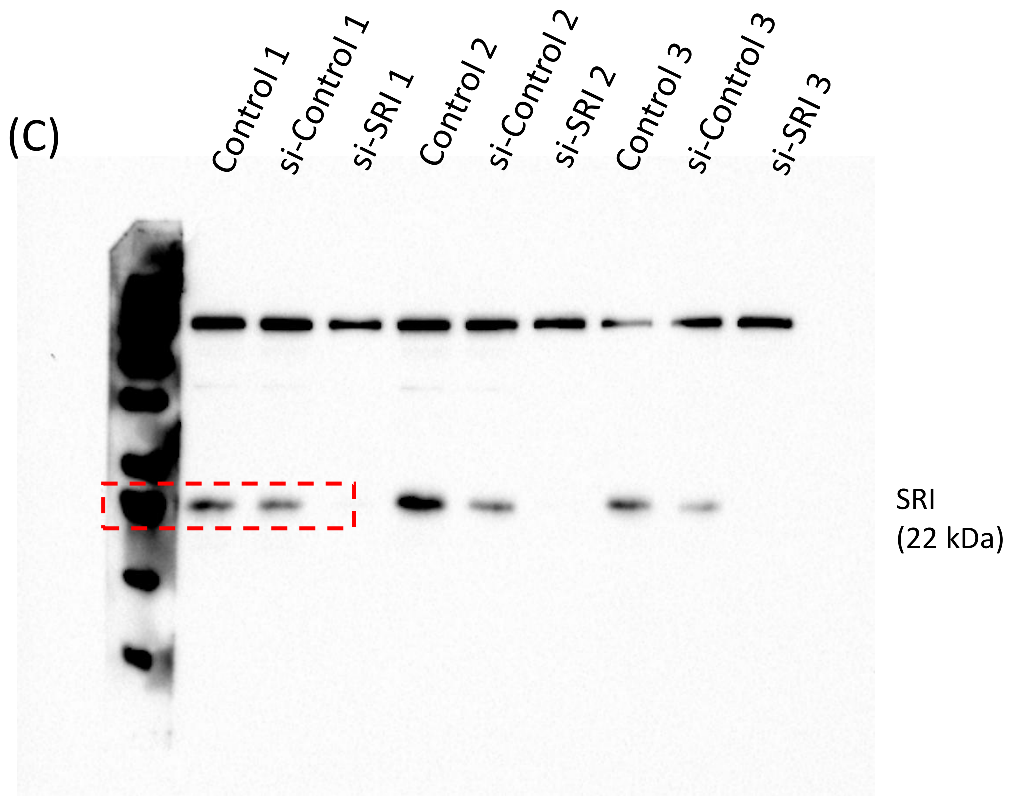

Supplementary Figure S1: Full-length blot images of Fig. 2 for the expression of SRI in the control, si-control and si-SRI. **(A)** SDS-PAGE image showing protein profile of cell lysate. Total density was used for normalization of protein load of cell lysate from control, si-control and si-SRI. **(B)** Western blot image showing expression of b-actin used as loading control. **(C)** Western blot image showing expression of SRI in control, si-control and si-SRI. The experiment was performed in triplicates. The cropping of the blot images is indicated with red dashed line.

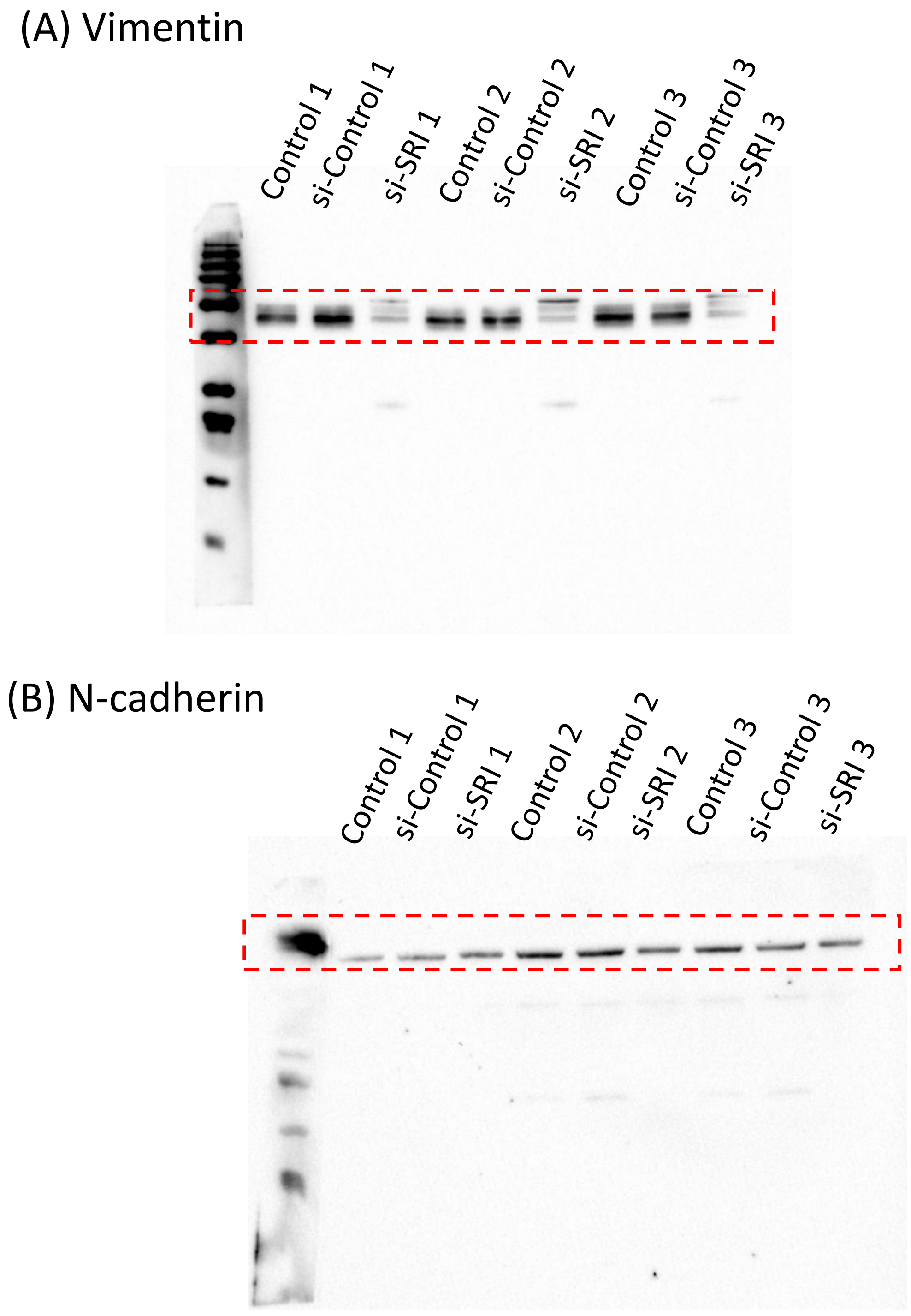

Supplementary Figure S2: Full-length blot images of Fig. 6 for the expression of **(A)** vimentin and **(B)** N-cadherin in the control, si-control and si-SRI. The experiment was performed in triplicates. The cropping of the blot images is indicated with red dashed line.
